# Parental age and offspring leukocyte telomere length and attrition in midlife: Evidence from the 1946 British birth cohort

**DOI:** 10.1016/j.exger.2018.09.008

**Published:** 2018-10-02

**Authors:** Wahyu Wulaningsih, Rebecca Hardy, Andrew Wong, Diana Kuh

**Affiliations:** MRC Unit for Lifelong Health and Ageing at UCL, London, UK

**Keywords:** Reproductive health, Parental age, Offspring health, Telomere, Ageing

## Abstract

**Background:**

There is evidence that paternal age may influence offspring telomere length, but the joint effects of father's and mother's age are unclear. We evaluated whether parental ages, individually and jointly, were associated with offspring telomere length and shortening.

**Methods:**

We included 2305 British birth cohort participants with measured leukocyte telomere length (LTL) at age 53, among whom 941 had a second measurement at age 60–64. Linear regressions were performed to assess the associations of father's and mother's age at birth and the parental age gap, i.e. the difference between maternal and paternal age with LTL and LTL change.

**Results:**

A one year increase in father's age corresponded to a 0.26% (95% CI: 0.04–0.47%) increase in offspring LTL at age 53 in the sex-adjusted model. No association was observed for mother's age. Associations of father's or mother's age with offspring LTL at age 53 went to opposite directions when both parental ages were included together. For the difference in parental age, every year that fathers were older than mothers corresponded to a 0.94% (95% CI, 0.38–1.50%) increase in LTL at age 53 after adjustment for potential confounders. Neither parental ages nor the difference in parental ages were correlated with LTL change.

**Conclusion:**

There was a joint effect of parental ages on offspring telomere length, further denoting a complex role of reproductive age in offspring health and ageing.

## 1 Introduction

Telomeres are the terminal part of the chromosomes containing tandem repeats of DNA sequences which maintain genomic stability (Blackburn, 2001). Telomere shortening is regarded as a hallmark of ageing and shorter leukocyte telomeres correlate to higher mortality risk in several studies (Huzen et al., 2014; Kimura et al., 2008). Telomere maintenance is determined by genetic and nongenetic factors from early life onwards (Charakida et al., 2014). Among early life factors, paternal age has been suggested as a determinant of adult telomere length, with approximately 15 to 20 base pairs longer leukocyte telomere length in the offspring for each year of paternal age at conception (Aviv and Susser, 2013).

It has been suggested that this association between paternal age and offspring telomere length may be biologically driven by elongation of sperm telomere length observed in older compared to younger men (Eisenberg, 2011). One potential explanation is that the number of sperm produced decreases with age, thereby the amount of telomerase, a key enzyme for telomere maintenance, which is divided for each remaining sperm would be greater with advancing age. The effect of maternal age is less consistent (Unryn et al., 2005; De Meyer et al., 2007). Potential biological explanations may include effects of maternal age on intrauterine stress and maternal hormonal status as these factors have been linked to telomere length in infants (Entringer et al., 2011; Entringer et al., 2015). There is a lack of knowledge of whether these associations remain when taking into account confounders such as socioeconomic position, and whether parental age influences the rate of telomere shortening. Additionally, since parental age may be correlated, associations between mother's age and telomere length may be influenced by father's age and vice versa. Therefore, it would be interesting to further assess the gap between father's and mother's age in relation to telomere length in offspring.

We investigated father's and mother's ages at birth and the difference between father's and mother's age at birth as potential predictors of leukocyte telomere length at age 53 and 60–64 in the Medical Research Council (MRC) National Survey of Health and Development (NSHD). Using two repeated measurements, we also examined telomere shortening to further gain insight into the role of parental reproductive age on offspring health and ageing.

## 2 Methods

### 2.1 Study population

The NSHD is based on a nationally representative sample of 5362 births out of all the single births to married mothers that occurred in one week in March 1946 in England, Scotland, and Wales. Details on the NSHD and its follow-up have been published elsewhere (Kuh et al., 2011). Ethical approval was obtained from the Greater Manchester Local Research Ethics Committee and the Scotland Research Ethics Committee. Written informed consent was obtained from the study members.

We included 2305 study members who had follow-up assessment at age 53 and complete information on both father's and mother's age at birth, father's occupation and telomere length. Of those, 941 had a second telomere length assessment at age 60–64. Parental age gap was calculated by subtracting mother's age from father's age.

### 2.2 Leukocyte telomere length

DNA was extracted from frozen EDTA blood samples using Puregene DNA isolation kits (Flowgen, Leicestershire, UK). Absolute leukocyte telomere length (LTL) was measured in the same laboratory according to a previously validated real-time polymerase chain reaction technique in a blinded fashion. Measurements were performed in quadruplicate on an Applied Biosystems 7900HT Fast Real Time PCR system with 384-well plate capacity. The intra-assay coefficient of variation was 2.7% while the inter-assay coefficient of variation was 5.1%. Internal DNA controls were used to normalise assays in different runs. For participants with repeated measurements, we calculated telomere attrition as the annual percentage change in LTL (ΔLTL) as follows: [(LTL_60–64_ − LTL_53_) × 100/LTL_53_)]/interval (years).

### 2.3 Potential confounders

Parental sociodemographic factors were considered potential confounders in the study. The Registrar General's social class classification was used as a measure of parental socioeconomic position (SEP), based on father's occupation when the study member was aged 4, and included 6 occupational classes ranging from unskilled (class V) to professional (class I; Table S1). In addition, mother's and father's highest levels of education were dichotomised into up to or higher than primary school. Region of place of residence at birth corresponded to the civil regions used in 1946. Information on whether a parent smoked when they lived with study members was reported by adult study members.

### 2.4 Statistical analysis

LTL was not normally distributed and was logarithmically transformed. We ran linear regression analyses with father's and mother's ages at birth included separately as predictors of log-transformed absolute LTL at age 53, age 60–64, or ΔLTL adjusted for sex of offspring (Model 1). We repeated the analysis for LTL at age 53 in the sample with second measurements of LTL at age 60–64. We further adjusted for other potential confounders including father's social class, father's and mother's education, region of residence, and parental smoking (Model 2).

To evaluate joint parental ages effect, we first included both father's and mother's age in the same model and adjusted for the same covariates (Model 3). The variance inflation factor was estimated to check whether there was multicollinearity between mother's and father's ages, with a value above 10 commonly regarded to reflect substantial collinearity (O'Brien, 2007). Secondly, we assessed parental age gap. This analysis was subsequently adjusted for father's age and then all covariates.

## 3 Results

Characteristics of study members contributing to the analysis of LTL at 53 and the change in LTL are presented as Table 1. On average, study members' fathers were 3 years (SD: 4 years) older than their mothers. Father's and mother's ages were highly correlated (*r* = 0.78, p < 0.001). Parental ages at birth and LTL at age 53 were similar between the two group who had one measurement and repeated LTL (Table 1). We compared LTL at age 53 between those who were still alive and those who had died by the time of second the measurement and found no difference (P_ttest_ = 0.9).

**Table 1:** Characteristics of study participants.

	All participants (N = 2162)	Participants with LTL measures at follow-up (N = 897)	Participants without LTL measures at follow-up (N = 1265)
Sex, male – N(%)	1075 (49.72)	426 (47.69)	649 (51.30)
Childhood socioeconomic position – N(%)			
Professional	139 (6.43)	63 (7.02)	76 (6.01)
Intermediate	434 (20.07)	203 (22.63)	231 (18.26)
Skilled (non-manual)	342 (15.82)	138 (15.38)	204 (16.13)
Skilled (manual)	707 (32.70)	282 (31.44)	425 (33.60)
Partly skilled	414 (19.15)	166 (18.51)	248 (19.60)
Unskilled	126 (5.83)	45 (5.02)	81 (6.40)
Father's education, higher than primary – N(%)	653 (30.20)	300 (33.44)	353 (27.91)
Mother's education, higher than primary – N(%)	475 (21.97)	214 (23.86)	261 (20.63)
Any parent smoked, yes – N(%)	851 (39.36)	334 (37.24)	517 (40.87)
Region – N(%)			
Scotland	235 (10.87)	105 (11.71)	130 (10.28)
Wales	122 (5.64)	60 (6.69)	62 (4.90)
Northern	171 (7.91)	85 (9.48)	86 (6.80)
East and West Ridings	154 (7.12)	94 (10.48)	60 (4.74)
North Western	228 (10.55)	146 (16.28)	82 (6.48)
North Midland	191 (8.83)	79 (8.81)	112 (8.85)
Midland	169 (7.68)	71 (7.92)	98 (7.75)
Eastern	166 (7.68)	55 (6.13)	111 (8.77)
London and South Eastern	486 (22.48)	131 (14.60)	355 (28.06)
Southern	111 (5.13)	24 (2.68)	87 (6.88)
South Western	129 (5.97)	47 (5.24)	82 (6.48)
Father's age at birth – Mean (SD), range	31.93 (6.40), 17–61	31.91 (6.30), 18–61	31.95 (6.47), 17–61
Mother's age at birth – Mean (SD), range	29.03 (5.66), 16–48	29.07 (5.68), 17–47	29.01 (5.65), 16–48
Parental age gap – Mean (SD)	2.90 (4.05)	2.85 (3.87)	2.94 (4.16)
LTL at age 53 (kbp) – Mean (SD)	5.66 (1.93)	5.68 (1.95)	5.65 (1.91)
LTL at age 60–64 (kbp) – Mean (SD)	–	4.30 (1.31)	–
Annual change in LTL (%) – Mean (SD)	–	−1.62 (4.49)	–

### 3.1 Father's age and LTL

In the full sample, every year increase in father's age corresponded to a 0.29% (95% CI: 0.05 to 0.49%) increase in offspring absolute LTL at age 53 in the sex-adjusted model. Associations were similar when additionally adjusted for potential confounders (Table 2, Model 2). The association between older father's age and longer LTL at 53 was slightly weaker when analysis was restricted to the subsample who had a second assessment at 60–64. The associations between greater father's age and longer LTL at age 60–64 and increases in ΔLTL were weak and the analysis failed to reject the null hypothesis of no association.

**Table 2:** Association between parental age at birth and absolute telomere length (LTL) at age 53 and 60–64 and with annual changes in telomere length between 53 and 60–64.

Per year age increase	Model 1			Model 2			Model 3		
	Percent difference	95% CI	p-value	Percent difference	95% CI	p-value	Percent difference	95% CI	p-value
Full sample (N = 2162)									
LTL at age 53 (%)									
Father's age	0.29	0.05 to 0.49	0.02	0.26	0.04 to 0.49	0.02	0.52	0.16 to 0.87	0.004
Mother's age	0.10	−0.14 to 0.35	0.82	0.09	−0.16 to 0.34	0.48	−0.37	−0.76 to 0.04	0.07

Sample with repeat LTL measures (N = 897)									
LTL at age 53^a^ (%)									
Father's age	0.38	0.03 to 0.73	0.03	0.33	−0.02 to 0.70	0.07	0.90	0.32 to 1.49	0.003
Mother's age	0.08	−0.31 to 0.47	0.67	−0.003	−0.40 to 0.40	0.98	−0.80	−1.44 to −0.14	0.02
LTL at age 60–64 (%)									
Father's age	0.09	−0.22 to 0.39	0.57	0.09	−0.21 to 0.40	0.55	0.16	−0.34 to 0.65	0.54
Mother's age	0.09	−0.24 to 0.42	0.52	0.05	−0.29 to 0.39	0.78	−0.09	−0.64 to 0.46	0.75
Annual change in LTL^b^ (% per year)									
Father's age	0.008	−0.02 to 0.04	0.63	0.007	−0.03 to 0.04	0.69	0.01	−0.04 to 0.07	0.69
Mother's age	0.008	−0.03 to 0.04	0.67	0.004	−0.03 to 0.04	0.85	−0.006	−0.07 to 0.05	0.84

Model 1: Adjusted for sex.

Model 2: Adjusted for sex and father's social class, father's education and mother's education, region, history of parental smoking.

Model 3: Father's and mother's age at birth included in the same model and adjusted for sex and father's social class, father's education and mother's education, region, history of parental smoking.

a Analysis limited to those with second measurements of LTL at age 60–64.

b Additionally adjusted for LTL at age 53.

### 3.2 Mother's age and LTL

There were no evidence of associations between mother's age and absolute LTL at age 53, in either the maximum or restricted sample, LTL at 60–64 or ΔLTL (Table 2, Models 1 and 2).

**Fig. 1:**
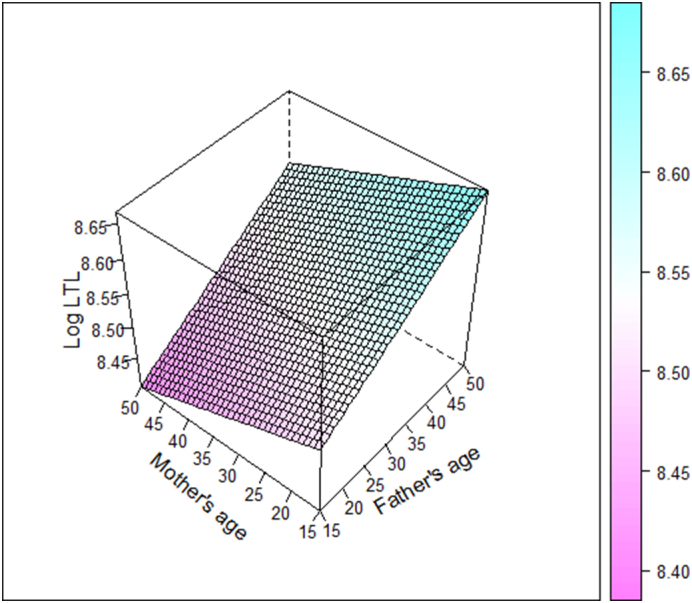
Joint effects of father's and mother's age at birth (years) on absolute leukocyte telomere length (LTL) at age 53, adjusted for sex and father's social class. The right-hand side colour legend indicates predicted values of log-transformed LTL.

### 3.3 Joint effects of parental age on LTL

In models including both father's and mother's age (Table 2, Model 3) the positive association between father's age and LTL at 53 became twice as much as the separate models and an inverse association between mother's age and LTL at 53 became evident although the 95% confidence interval included 0 (the null value). In the subgroup with LTL data at both age 53 and 60–64, the inverse association between mother's age and LTL was particularly strong with a 0.80% decrease (95% CI: 0.14 to 1.44%) in absolute LTL with every year increase in mother's age. The variance inflation factor between father's and mother's age was 2.69, suggesting that multicollinearity was not a problem. The joint effect of father's and mother's age is further depicted in Fig. 1, showing their opposing effect on LTL at age 53. The same trend was shown when we used parental age gap as the predictor, in which every year father was older than mother corresponded to 0.49% (95% CI: 0.13 to 0.83%) longer LTL at age 53 (Table S1, model 2).

## 4 Discussion

In the NSHD, offspring of older fathers had longer LTL at age 53, whereas no association was observed with mother's age. When we took into account both father's and mother's age, the positive association between father's age and LTL became stronger and there was a weak inverse association between mother's age and LTL at age 53 in the subgroup who had repeated LTL measures, meaning that a greater difference between parental ages was also associated with LTL at 53. No association between either father's or mother's age with LTL at age 60–64 or telomere attrition.

Our results on father's age at birth and LTL corroborated prior findings such as a study of 5127 individuals aged 15 to 89 across five European cohorts which showed telomere length to be positively correlated with paternal age (Broer et al., 2013). This association between paternal age and offspring telomere length was further shown to be already present among newborns (Factor-Litvak et al., 2016) and to persist across two generations (Eisenberg et al., 2012). We added to this evidence by assessing the association between father's age and rate of telomere change and by considering the parental age difference. We also included different socioeconomic status and smoking history and showed that the positive association between father's age and LTL remained after taking into account potential confounders.

We found opposing effect between father's and mother's age on offspring LTL at age 53 when they were jointly assessed. Having a younger mother, conditional on father's age, corresponded to longer LTL at age 53 particularly in the subgroup with LTL measured at both 53 and 60–64. Adjusting for father's age also revealed a weak inverse trend between maternal age and LTL in the Nurses' Health Study (Prescott et al., 2012) and a European study (Broer et al., 2013). However, our findings on this joint effect and parental age gap suggest a complex interplay between father's and mother's age in the context of offspring telomere length. This finding may have been explained by the correlation between father' and mother's ages. Longer LTL with greater father's age may be explained by the decrease in sperm count and higher telomerase content per sperm (Eisenberg, 2011). On the other hand, declining numbers and quality of ovum with older age of mothers may negatively influence offspring health. This was supported by improvement in quality of fertilised oocytes and early embryo development upon antioxidant treatment to delay such ovarian ageing (Liu et al., 2012). Therefore, in the context of the ageing process in offspring, it might be more useful to evaluate the effects of both father's and mother's age rather than assessing them individually.

Our cohort was based on singleton born in a single week to married parents, however, the study was broadly representative of native-born British men and women of the same age (Stafford et al., 2013). Since study members were all born during the same period, this study may be less affected by increasing or decreasing gap between father's and mother's age across generations. We included two repeated assessments of LTL which allowed analysis of telomere attrition. Our study was limited by cohort attrition and relatively small sample size. Although there was no difference in terms of LTL at 53 among those who died and those who were followed up, survivor bias may still have affected the study. Thereby, our findings in the subsample completing both assessments may point towards specific mechanisms linking parental ages or age gap and telomere maintenance among those with better health in older age. We obtained parental smoking status retrospectively since data at birth was not available. Another limitation was the use of circulating LTL which may correlate less with specific diseases compared to tissue-based LTL. Despite consistencies across studies on the association between paternal age and telomere length, further research in a bigger population or across populations with greater variation in parental age differences is needed.

## 5 Conclusion

Our study supports the positive association between father's age and offspring LTL at age 53 but not 60–64, and suggests a role for a large difference in parental age on offspring LTL. As circulating LTL has often been considered a marker of ageing, these findings suggest the need to further assess the impact of reproductive age on offspring health and ageing outcomes.

The following is the supplementary data related to this article.

Table S1 Association between parental age gap (per year difference) at birth and absolute telomere length (LTL) at age 53 and 60–64 and with annual changes in telomere length between 53 and 60–64. Table S1

## Funding

This work is supported by the UK Medical Research Council which provides core funding for the MRC National Survey of Health and Development and W.W. R.H. A.W. and D.K. [MC_UU_12019/1; MC_UU_12019/2; MC_UU_12019/4].
