# Supplementary material for: Parental age and offspring leukocyte telomere length and attrition in midlife: Evidence from the 1946 British birth cohort
Source: Exp Gerontol. 2018 Oct 2;112:92–6. doi: 10.1016/j.exger.2018.09.008 (PMC6189452; doi:10.1016/j.exger.2018.09.008)
Supplement: Table S1 — Association between parental age gap (per year difference) at birth and absolute telomere length (LTL) at age 53 and 60–64 and with annual changes in telomere length between 53 and 60–64. [file mmc1.docx]

**Table S1**. Association between parental age gap (per year difference) at birth and absolute telomere length (LTL) at age 53 and 60-64 and with annual changes in telomere length between 53 and 60-64.

|  | **Model 1** | | | **Model 2** | | | **Model 3** | | |
| --- | --- | --- | --- | --- | --- | --- | --- | --- | --- |
|  | **Percent difference** | **95% CI** | **p-value** | **Percent difference** | **95% CI** | **p-value** | **Percent difference** | **95% CI** | **p-value** |
| **Full sample (N=2162)** | |  |  |  |  |  |  |  |  |
| **LTL at age 53** |  |  |  |  |  |  |  |  |  |
| Parental age gap | 0.47 | 0.12 to 0.82 | 0.008 | 0.49 | 0.13 to 0.83 | 0.007 | 0.37 | -0.04 to 0.77 | 0.07 |
| **Sample with repeat**  **LTL measures (N=897)** | |  |  |  |  |  |  |  |  |
| **LTL at age 53^a^** |  |  |  |  |  |  |  |  |  |
| Parental age gap | 0.84 | 0.27 to 1.40 | 0.004 | 0.87 | 0.30 to 1.45 | 0.003 | 0.80 | 0.14 to 1.44 | 0.02 |
| **LTL at age 60-64** |  |  |  |  |  |  |  |  |  |
| Parental age gap | 0.04 | -0.45 to 0.53 | 0.87 | 0.14 | -0.35 to 0.62 | 0.58 | 0.08 | -0.46 to 0.64 | 0.75 |
| **Annual change in LTL** |  |  |  |  |  |  |  |  |  |
| Parental age gap | 0.005 | -0.05 to 0.06 | 0.87 | 0.01 | -0.04 to 0.06 | 0.72 | 0.006 | -0.05 to 0.78 | 0.84 |

Model 1: Adjusted for sex

Model 2: Adjusted for sex and father’s occupational social class

Model 3: Adjusted for sex, father’s occupational social class and father’s age

^a^Analysis limited to those with second measurements of LTL at age 60-64

^b^Additionally adjusted for LTL at age 53
